# CpG-Oligodeoxynucleotide Treatment Protects against Ionizing Radiation-Induced Intestine Injury

**DOI:** 10.1371/journal.pone.0066586

**Published:** 2013-06-21

**Authors:** Chao Zhang, Jin Ni, Bai-Long Li, Fu Gao, Hu Liu, Wen Liu, Yi-Juan Huang, Jian-Ming Cai

**Affiliations:** Section of Radiation Medicine, Department of Naval medicine, Second Military Medical University, Shanghai, China; Northwestern University Feinberg School of Medicine, United States of America

## Abstract

**Background:**

the bone marrow and the intestine are the major sites of ionizing radiation (IR)-induced injury. Our previous study demonstrated that CpG-oligodeoxynucleotide (ODN) treatment mitigated IR-induced bone marrow injury, but its effect on the intestine is not known. In this study, we sought to determine if CpG-ODN have protective effect on IR-induced intestine injury, and if so, to determine the mechanism of its effect.

**Methods and Findings:**

Mice were treated with CpG-ODN after IR. The body weight and survival were daily monitored for 30 days consecutively after exposure. The number of surviving intestinal crypt was assessed by the microcolony survival assay. The number and the distribution of proliferating cell in crypt were evaluated by TUNEL assay and BrdU assay. The expression of Bcl-2, Bax and caspase-3 in crypt were analyzed by Immunohistochemistry assay. The findings showed that the treatment for irradiated mice with CpG-ODN diminished body weight loss, improved 30 days survival, enhanced intestinal crypts survival and maintained proliferating cell population and regeneration in crypt. The reason might involve that CpG-ODN up-regulated the expression of Bcl-2 protein and down-regulated the expression of Bax protein and caspase-3 protein.

**Conclusion:**

CpG-ODN was effective in protection of IR-induced intestine injury by enhancing intestinal crypts survival and maintaining proliferating cell population and regeneration in crypt. The mechanism might be that CpG-ODN inhibits proliferating cell apoptosis through regulating the expression of apoptosis-related protein, such as Bax, Bcl-2 and caspase-3.

## Introduction

The small intestinal epithelium is continuously and rapidly replaced by cells proliferation within the crypts and the subsequent migration of their progeny to the villous epithelium. Intestinal epithelial cells are ultimately derived from these proliferating cell [1]. Although the multiple chemical, physical, infectious and inflammatory agents could cause intestine injury, ionizing radiation (IR) is the most extensive factor for intestine injury, which affects the rapidly proliferating cell population. In small intestine, there is a significant amount of proliferating cell located within crypt, including intestinal stem cells and proliferative crypt cells. Intestinal stem cell is thought to occupy a niche located at approximately position 4 above crypt base. The distribution of proliferative crypt cell present in each crypt remains a subject of speculation (cell position from 5 to 16, approximately). The stem cell and its immediate descendants, proliferative crypt cell, are arranged in a functional hierarchy, where hierarchical position is related to topologic position in the crypt [2]. When mice were exposed to high dose of IR, a large number of proliferating cell located within the intestinal crypt are killed, except fewer survival proliferating cells [3]. These surviving proliferating cells play a central role in the regeneration of the intestinal crypt. They proliferate, form regenerative crypts and eventually repopulate the entire epithelium. With higher doses of IR, the number of surviving proliferating cells may be insufficient to regenerate the crypt, and consequently the number of crypt decrease [1]. Loss of these crypts after IR prevents normal re-epithelialization of the intestinal epithelium, which leads to different degrees of villous fusion and attenuation, as well as intestinal epithelial cells hypertrophy. These changes result in the acute radiation-induced gastrointestinal syndrome (RIGS) presenting malabsorption, electrolyte imbalance, diarrhea, weight loss and death. Therefore, the development of agents for prevention or treatment of IR-induced intestine injury has primarily focused on the protection of intestinal crypt, the mechanism responsible for the maintenance of proliferating cell population and regeneration.

Many studies indicated that bacterial products could affect the intestinal epithelial cellular response to IR injury. Toll-like receptors (TLRs) were found to play an essential role. These receptors recognize pathogen-associated molecular patterns (PAMPs) associated with pathogens [4], mediate interaction between bacterial production and intestinal epithelium, and influence the intestinal epithelial cellular response to IR injury [5]. Different reports have showed that lipopolysaccharide (LPS), a TLR4 ligand, is a radioprotective agent for mice intestine tissue through a prostaglandin E2 (PGE2)-mediated mechanism [1]. Probiotic, a TLR2 ligand, also has similar effect and mechanism [5]. Flagellin or CBLB502, a TLR5 ligand, may reduce the intestine IR injury by NF-κB activation [6], [7]. However, due to toxicity and other issues, these ligands are limited to use as a clinical agent.

CpG-oligodeoxynucleotide (ODN), unmethylated short single stranded synthetic DNA molecules, is a TLR9 ligand, which does not present know adverse effects and has been clinically evaluated and used as vaccine adjuvants and antitumor immunotherapy agent [8]–[10]. Our previous study found that CpG-ODN mitigated IR-induced bone marrow injury. In experimental models of IR injury, CpG-ODN given to mice resulted in the inhibition of white blood cell (WBC) apoptosis and enhancement of the bone marrow cell recovery [11]. Recently, G.Pedersen et al and Jongdae L et al reported that CpG-ODN by TLR9 in intestinal epithelium contributed to intestinal homoeostasis [12], [13]. Preliminary studies of CpG-ODN indicate that it prevents IR injury in the intestine [14]. However, no information is available on the treatment effect of CpG-ODN on the intestine IR injury, nor any mechanism was described. In the present, our purpose was to determine if CpG-ODN treats IR-induced intestine injury, and if so, to determine the mechanism of its effect.

## Materials and Methods

### Reagent

CpG-ODN was synthesized at Shanghai Sangon Biological engineering Technology Services Co Ltd (Sangon, China). CpG-ODN sequences used in this study were 5′-T***CG*** T***CG*** TTT T***CG*** GC***G*** *****CG***C ***C***G-3′ (regular letters represent phosphorothioate, bold and italic letters represent phosphodiester) [15]. The compound was diluted with phosphate buffer saline (PBS) to a final concentration of 250 µg/ml and stored at 4°C.

### Animals

Male BALB/c mice (19∼21 g), 6∼7 weeks old, were purchased from SLAC laboratory animal Co. Ltd (SLAC, China), and were kept under standard laboratory conditions (a temperature of 23±2°C with 24-hour cycles of fresh air and 12 h light/dark cycle). Food and water were sterilized by ^60^Co γ-radiation and high pressure respectively. All protocols were approved by the Second Military Medical University (SMMU, China), in accordance to the Guide for Care and Use of Laboratory Animals published by US NIH (publication No 96∼101).

### Treatment

Mice were treated with either 0.2 ml CpG-ODN (250 µg/ml) or 0.2 ml PBS via intraperitoneal (i.p) injection. CpG-ODN was given 30 min, 24 h and 48 h after IR, unless stated otherwise. Mice were divided into four groups as follows: a normal group (the non-irradiated mice with PBS treatment), a CpG-ODN group (the non-irradiated mice with CpG-ODN treatment), a IR control group (the irradiated mice with PBS treatment) and a IR plus CpG-ODN group (the irradiated mice with CpG-ODN treatment).

### Irradiation

Irradiation was done with the ^60^Co γ-radiation at SMMU facility. Each mouse was placed inside a specially designed and well-ventilated acrylic container with dimensions of 8.0 × 3.5 × 3.5 cm. The mice were then subjected to 8 Gy total body irradiation under standard laboratory conditions. The dose rate was 0.8 Gy/min.

### Survival and Body Weight

Mice were treated with CpG-ODN after IR. To determine the survival and body weight, daily monitoring was performed for 30 days. Survival was expressed as percent survival. Average body weight was calculated considering the initial body weight of the animal as 100%.

### Histological Assessment

Mice were euthanized by cervical dislocation at 0.5, 1, 2, 3, 5, 7 days after IR. The appropriate segments of the small intestine were rapidly excised, rinsed with PBS, and pinned on a corkboard. Portions of the specimen were fixed for 48 h in 10% neutral buffered formalin. Slides 5 µm-thick were prepared and stained with hematoxylin and eosin (H&E).

### Measuring and Scoring

The microcolony survival assay is a reproducible method of assessing injury to the intestinal crypt and has been widely used since it was first described in 1970 by Withers and Elkind [16] **.** The number of surviving crypts per circumference was counted in 3 sections per mice, using a Nikon ECLIPSE microscope (a 400× magnification was applied). Only transversely sectioned crypts of 10 or more epithelial cell (excluding Paneth cells) were counted. Crypts consisting of less than 10 non-Paneth cells were considered to have no surviving crypt, and not to contribute to the repopulation of the epithelium. The width of all longitudinally crypts per circumference was also measured under microscope with the aid of a micrometer graticule (5 mm/100 points, Shanghai optical instrument factory, Shanghai, China) placed in an eyepiece with a 10 × magnification. The width of each crypt was measured at the widest point with a 40 × magnification. Because of the variation due to different treatments, the mice crypt diameters were corrected according to the factor D*c*/D*t*, where D represents the crypt transverse diameter, *c* is normal and *t* is treated. The number of surviving crypts per circumference for each group was obtained by multiplying the counted crypt by the appropriate correction factor [17].

### TUNEL Staining

Apoptotic cells were determined by terminal deoxynucleotidyl transferase-mediated deoxyuridine triphosphate nick end labeling (TUNEL) staining. Mice were euthanized 5 hours after IR. Small intestine was rapidly excised, rinsed with PBS, pinned on a corkboard, and fixed in 10% neutral buffered formalin. Portions of the specimen were paraffin embedded and 5 µm sections were cut. Sections were deparaffinized, permeabilized with proteinase K (20 µg/ml, Sigma) at 23°C and rinsed 4 additional times with distilled water followed by incubation with a solution made up of TdT (1 µl/200µl of mix solution), bovine serum albumin (1 mg/ml), and biotin-16-dUTP (1 nmol/50 µl of mix solution) in TdT buffer. Sections were then incubated in saline citrate for 15 min, rinsed and incubated with 2% bovine serum albumin at 23°C. Endogenous peroxidase activity was blocked by incubating the tissue sections with 3% H_2_O_2_. The labeled DNA fragments were detected with peroxidase-conjugated antibody elicited against biotin. Slides were developed with DBA (diaminobenzidine tetrahydrochloride), and counted after stained with hematoxylin.

### BrdU Labeling

Proliferative cells were determined by BrdU incorporation. S phase cells were labeled in vivo by administering BrdU (i.p, 2.5 mg/mice, Sigma, USA) 2 hours before euthanasia. Mice were euthanized 3 days after IR. Small intestine was rapidly excised, rinsed with PBS, pinned on a corkboard, and fixed in 10% neutral buffered formalin, and embedded in paraffin. Sections (5 µm) of paraffin embedded small intestine were deparaffinized and incubated with 3% H_2_O_2_ in methanol for 15 min. Sections were incubated with 2N HCl for 1 h, washed in PBS, and then incubated in 0.1% trypsin for 15 min at 37°C. Sections were stained for BrdU incorporation using a BrdU staining kit (Cat.No.KT-077, Kamiya Bioedical Company, California, USA) according to the manufacturer instructions.

### Immunohistochemistry

Bal-2, Bax and Caspase-3 were measured by immunohistochemistry. Mice were euthanized 6 hours and 48 hours after IR [18]. Small intestine was rapidly excised, rinsed with PBS, pinned on a corkboard. Following fixation, the tissue was paraffin embedded and 5 µm sections were cut. Immunohistochemistry was performed on paraffin-embedded sections. The primary antibodies used were mouse monoclonal anti-Bcl-2 (C-2) by ZSGB-Bio (ZS-7382) dilution 1∶50; mouse monoclonal anti-Bax (B-9) by Santa Cruz (sc-7480) dilution 1∶100; and rabbit polyclonal anti-caspase-3 (H-277) by ZSGB-Bio (ZS-7148) dilution 1∶50. All primary antibodies were applied overnight at 4°C. Secondary antibodies were either biotin-labeled goat anti-rabbit immunoglobulin (Santa Cruz, SP9001) or bio-labeled goat anti-mouse immunoglobulin (Santa Cruz, SP9002) at a dilution of 1∶100 for 30 min at room temperature.

### Positional Analysis

The intestinal crypts per circumference were scored on a cell positional basis for labeled cells as previously described in detail for the small intestine by Pritchard D.M for the small intestine [19]. Cells were numbered from the base of the crypt, designated cell position 1, and scored as labeled cells, up to the crypt-villus junction. Data are presented as plots of labeled cells index percentage against cell position along the crypt and as mean labeled cells index percentage for each mice group.

### Statistical Analysis of Data

For the survival data, the difference in 30 days survival between was analyzed by Kaplan-Meier plots. For body weight, crypt survival, positional labeled cell and the number of labeled cell, the difference between IR plus CpG-ODN group and IR control group was analyzed by the two-sided, non-paired Student’s *t-*test. Data from all the experiments are expressed as *mean* ± standard error of mean (SEM). The software analysis program SPSS 13.0 (Release 12.0 K; SPSS Inc., Chicago, USA) was used. All differences were considered significant if *p* was less than 0.05.

## Results

### CpG-ODN Diminishes Weight Loss and Improves Survival after IR

**Figure 1:**
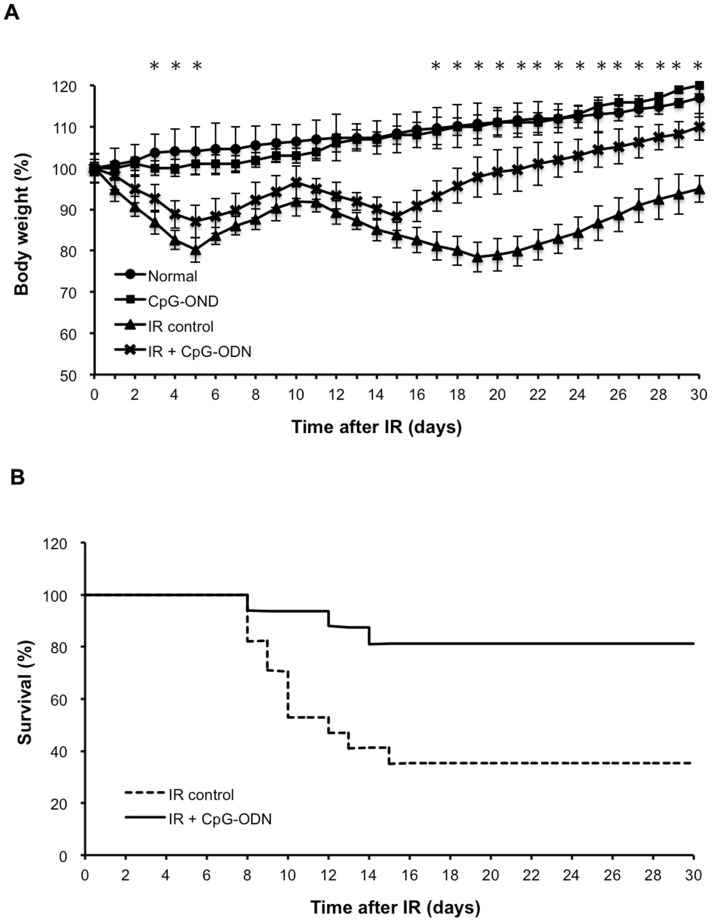
CpG-ODN diminishes weight loss and improves survival after IR. Mice were treated with CpG-ODN after 8 Gy of IR. Body weight (A) and survival (B) were monitored for 30 days. (n = 8/group in each of three separate experiments. Data are represented by mean ±SEM. the difference in 30 days survival between the two groups was analyzed by Kaplan-Meier plots. *p<0.05 for differences between IR plus CpG-ODN and IR control groups).

Mice exposed to 8 Gy produce a characteristic RIGS comprising weight loss and death [3]. This process is divided in four phases: prodromal, latent, critical and recovery [20]. In the prodromal, mice consistently lose body weight. In the latent, they begin to regain weight and reach a plateau. In the critical, mice begin to loss weight again. In the recovery, body weight slowly returns to normality. Irradiated group treated with CpG-ODN showed an increase in the body weight, when compared with irradiated control group in all phases, especially in the prodromal (from 4 to 6 days after IR) and recovery phases (from 16 to 30 days after IR). Furthermore, treating with CpG-ODN after IR injury, body weight came back to normal earlier than that of irradiated control group (fig. 1A). Death of exposed animal is a typical endpoint in the study of IR injury and described as the most attributable consequence to the combined effects of RIGS and bone marrow failure. Although irradiated mice in the groups either treated with or without CpG-ODN began to die after 9 days of IR, the 30 days survival of the CpG-ODN treated group increased 46% as compared with the control one (fig. 1B). These results suggest that CpG-ODN decreased mice weight loss and improved the survival after IR injury.

### CpG-ODN Enhances Intestinal Crypts Survival after IR

**Figure 2:**
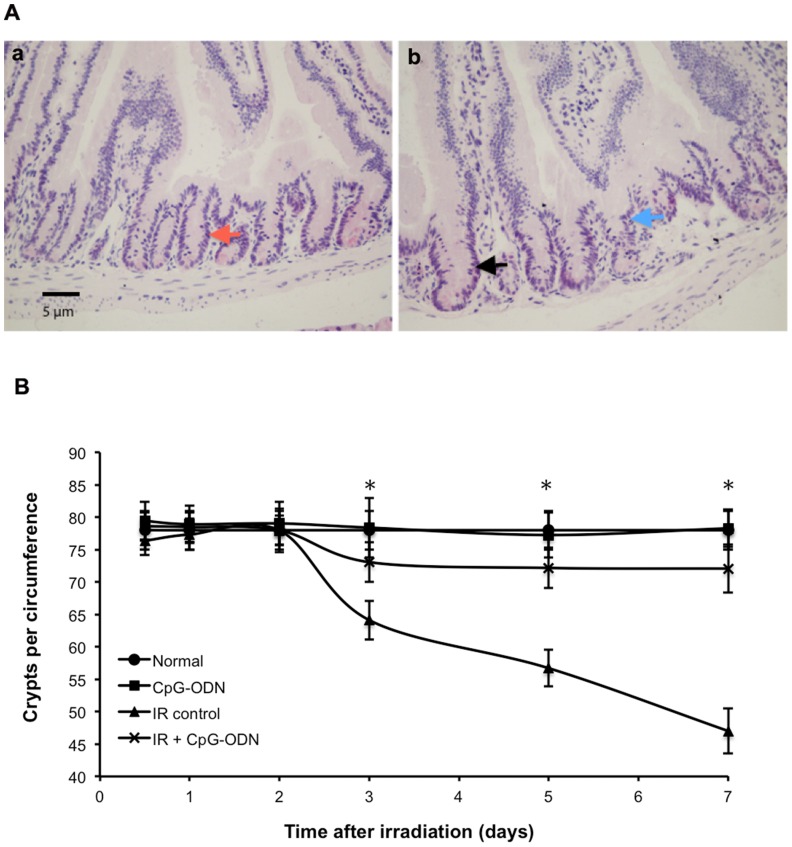
CpG-ODN enhances intestinal crypt survival after IR. Mice were treated with CpG-ODN and euthanized at indicated time points after 8 Gy of IR. The appropriate segments of small intestine were obtained and H&E stained. (A) Effects of IR on small intestinal crypt. Representative histological specimens of the small intestine are showed. (a) The appearance of a transverse section of the small intestinal normal crypt (red arrow); (b) the effect of IR in the small intestinal crypt at 7 days after IR, the number of crypts is notably reduced, the size of some crypts beyond normal value (black arrow), and some crypts disappear (blue arrow). (B) CpG-ODN enhances crypt survival. The number of surviving crypts per circumference was scored at 0.5, 1, 2, 3, 5 and 7 days after IR (n = 3/group in each of three separate experiments, with 3 sections per mice. Data are represented by the mean ±SEM. *p<0.05 for differences between IR plus CpG-ODN and IR control groups).

The microcolony assay is a reproducible method of assessing IR injury to the crypt. Mice exposed to 8 Gy IR crypt cell was death. In an attempt to compensate for the reduced input of cells from crypt to the epithelium, some crypts with surviving proliferative cell increase their size beyond normal value (fig. 2A). Because of variations in the crypt diameter of mice subjected to different treatment affect the crypt count, a correction for small differences in the crypt size of each group was described. The number of surviving crypts per circumference evaluated considering the correction factors applied (Table 1). The results showed that IR caused a significant and continual reduction in the number of surviving crypt 3 days after IR. However, the irradiated group treated with CpG-ODN, the number of surviving crypts was significantly larger than that of irradiated control group 3 days after IR. Especially on day 7, CpG-ODN treatment increased the number of surviving crypts from 60% to 92% (fig. 2B). These suggest that CpG-ODN enhanced intestinal crypts survival after IR injury.

**Table 1:** Number of surviving intestinal crypt after IR.

Time		Group	Crypts per circumference(uncorrected)	Crypts diameter (µm)	Correction factor	Crypts per circumference (corrected)
	Normal		78.0±9.0	41.1±8.6	1.000	78.0±9.0
0.5 d	CpG-ODN		79.0±6.0	40.9±10.8	1.005	79.4±6.0
	IR		76.0±7.2	40.9±8.1	1.005	76.4±7.2
	IR+CpG-ODN		77.0±5.1	40.4±7.8	1.017	78.6±5.2
1 d	CpG-ODN		79.3±7.9	41.3±7.6	0.995	78.9±7.9
	IR		74.7±6.4	39.7±7.1	1.035	77.3±6.6
	IR+CpG-ODN		76.7±5.3	39.8±7.3	1.033	78.5±5.5
2 d	CpG-ODN		80.0±5.3	41.6±6.7	0.988	79.0±5.2
	IR		71.7±6.4	37.8±7.2	1.087	78.0±7.0
	IR+CpG-ODN		75.0±5.3	39.0±7.6	1.054	78.0±5.6
3 d	CpG-ODN		80.3±9.6	42.1±9.6	0.976	78.4±9.4
	IR		66.0±7.0	42.3±9.3	0.972	64.1±6.8
	IR+CpG-ODN		72.0±6.1	40.5±7.3	1.015	73.1±6.2*
5 d	CpG-ODN		79.7±8.5	42.4±8.6	0.969	77.3±8.2
	IR		59.6±7.8	43.2±5.7	0.951	56.7±7.4
	IR+CpG-ODN		74.7±6.1	41.4±5.9	0.993	72.2±6.1
7 d	CpG-ODN		80.0±5.9	42.0±7.9	0.979	78.3±5.8*
	IR		56.7±6.5	45.2±8.5	0.909	47.0±5.9
	IR+CpG-ODN		78.0±7.7	42.2±9.3	0.974	72.1±7.5*

(n = 3/group in each of three separate experiments, with 3 section per mice; Data are the mean ±SEM.

* p<0.05 for the diffencers between IR plus CpG-ODN group and IR control group).

### CpG-ODN Maintains Proliferating Cell Population and Regeneration after IR

**Figure 3:**
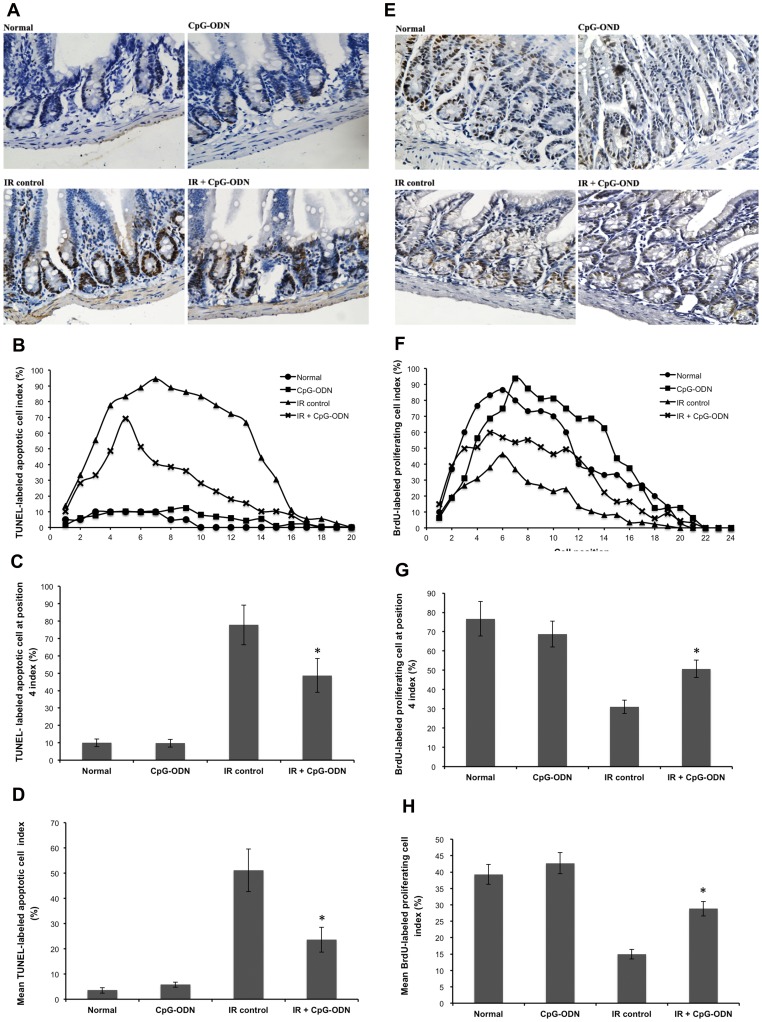
CpG-ODN maintains proliferating cell population and regeneration after IR. Mice were treated with CpG-ODN and euthanized at 5 hours after 8 Gy of IR. Samples were assessed by TUNEL assay. (A) Representative photomicrographs showing TUNEL-labeled apoptotic cell, (B) positional distribution of TUNEL-labeled apoptotic cell, (C) the number of TUNEL-labeled apoptotic at position 4, and (D) the mean of TUNEL-labeled apoptotic cell. Mice were treated with CpG-ODN and euthanized at 3 days after 8 Gy of IR. Samples were assessed by BrdU assay. (E) Representative photomicrographs showing BrdU-labeled proliferative cell, (E) positional distribution of BrdU-labeled proliferating cell, (F) the number of BrdU-labeled proliferating cell at position 4, and (G) the mean of BrdU-labeled proliferative (n = 3/group in each of three separate experiments, with 3 section per mice, data are represented by the mean ±SEM. *p<0.05 for the differences between IR plus CpG-ODN and IR control groups).

Proliferating cells, including intestinal stem cells located at position 4 above crypt base and proliferative crypt cells located in position 5∼16, play an important role in forming the crypt [21]. In order to determine whether CpG-ODN could maintain these proliferating cells population and regeneration after IR, the distribution and the number of their apoptosis and proliferative ability were evaluated by TUNEL and BrdU assays respectively. Firstly, we found that CpG-ODN inhibited proliferating cell apoptosis after 8 Gy of IR (fig. 3A). The distribution results showed that in the irradiated control group, the apoptotic cell index was >50% from position 3∼13. CpG-ODN treatment resulted in a significant index reduction. The apoptotic cell index was >50% only from positions 5∼6 (fig. 3B). Furthermore, CpG-ODN inhibited cell apoptosis at position 4 from 77.8% to 48.7% (fig. 3C). Position 4 is the position of intestinal stem cell that plays a key role in the regeneration of the crypts after IR exposure [18]. The result showed a 2.2-fold decrease in the number of apoptotic cell in irradiated group with CpG-ODN treatment compared with irradiated control group (fig. 3D). Secondly, CpG-ODN promoted proliferating cell regeneration (fig. 3E). The proliferating cell distribution showed that in irradiated control group, the proliferating cell index was higher than 50% at any position of irradiated control mice. CpG-ODN treatment resulted in a significant promotion as the proliferating cell index was >50% in position 4∼9 (fig. 3F). CpG-ODN promoted cell proliferation at position 4 from 31.0% to 50.8% (fig. 3G). The result showed a 1.9-fold increase in the number of proliferating cell in the irradiated CpG-ODN treated group compared with the irradiated control group (fig. 3H). These results suggest that CpG-ODN maintained the population and regeneration of proliferating cell located within the crypt after IR injury.

### CpG-ODN Regulates the Expression of Bax and Bcl-2 Expression after IR

**Figure 4:**
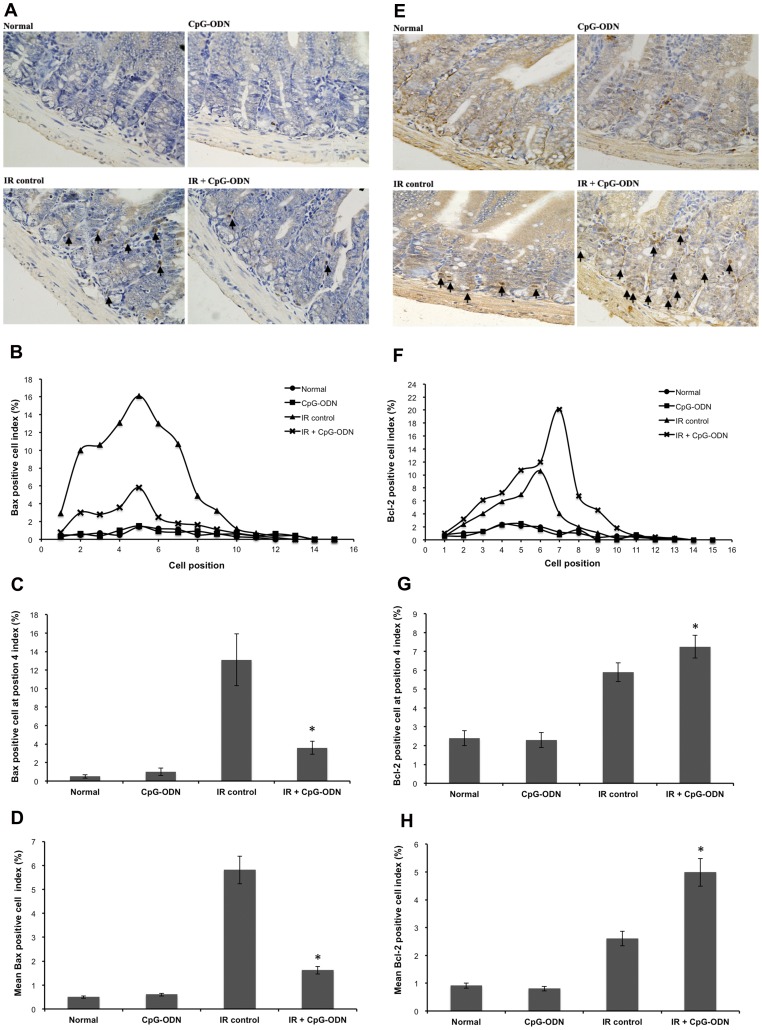
CpG-ODN regulates the expression of Bax and Bcl-2 after IR. Mice were treated with CpG-ODN and euthanized at 6 hours after 8 Gy of IR. Samples were detected by immunohistochemistry. (A and E) Representative photomicrographs showing Bax positive cell and Bcl-2 positive cell (arrowed), (B and F) positional distribution of Bax positive cell and Bcl-2 positive cell, (C and G) the number of Bax positive cell and Bcl-2 positive cell at position 4, (D and G) the number of mean Bax positive cell and Bcl-2 positive cell. (n = 3/group in each of three separate experiments, with 3 section per mice, data are represented by the mean ±SEM. *p<0.05 for the diffencers between IR plus CpG-ODN and IR control groups).

To describe the underlying mechanism of CpG-ODN, we investigated the expression of Bax and Bcl-2 proteins as the prominent members among Bcl-2 family. Mice were exposed to 8 Gy. Bax expression in crypt showed a position-dependent fashion (fig. 4A). The positive Bax cell index in exposed untreated mice was >5% at positions 2∼7. The index reached a maximum of 16.1% at position 5. For exposed mice treated with CpG-ODN, Bax positive cell index was >5% only at position 5. The index reached a maximum of 5.8% at position 5 (fig. 4B). CpG-ODN also down-regulated the number of Bax positive cell at position 4 from 13.1% to 3.6% (fig. 4C). The number showed a 3.6-fold decrease in the number of Bax positive cell for the irradiated group with CpG-ODN treatment compared with the irradiated control group (fig. 4D). In view of the expression of Bax, the expression of Bcl-2 was measured. The Bcl-2 expression also showed a position-dependent fashion (fig. 4E). The result of distribution showed that in irradiated control group, Bcl-2 positive cell index was >5% at positions 4∼6. The index reached a maximum of 10.6% at position 6. For exposed mice treated with CpG-ODN, a significant enhancement in position dependence was detected, Bcl-2 positive cell index was >5% at positions 3∼8. The index reached a maximum of 20.1% at position 7 (fig. 4F). CpG-ODN also up-regulated the number of Bcl-2 positive cell at position 4 from 5.9% to 7.3% (fig. 4G). The result showed that a 2-fold increase in the number of Bcl-2 positive cell for the irradiated group with CpG-ODN treatment compared with the irradiated control group (fig. 4H). These results suggest CpG-ODN down-regulated Bax expression and up-regulated Bcl-2 expression in a position-dependent fashion after IR injury.

### CpG-ODN Regulates the Expression of Caspase-3 after IR

**Figure 5:**
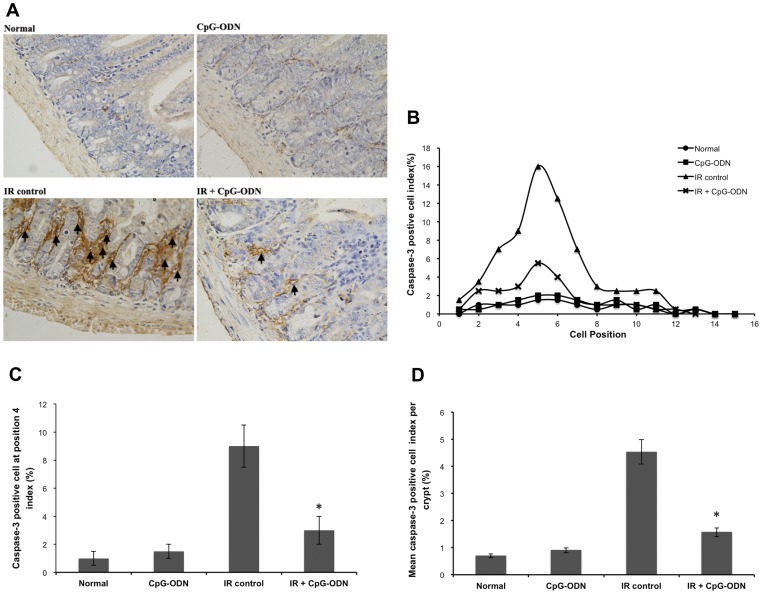
CpG-ODN regulates the expression of caspase-3 after IR. Mice were treated with CpG-ODN and euthanized at 48 hours after 8 Gy of IR. Samples were assessed by immunohistochemistry. (A) Representative photomicrographs showing caspase-3 positive cell (arrowed), (B) positional distribution of caspase-3 positive cell, (C) the number of caspase-3 positive cell at position 4, and (D) the mean of caspase-3 positive per crypt. (n = 3/group in each of three separate experiments, with 3 section per mice, data are represented by the mean ±SEM. *p<0.05 for differences between IR plus CpG-ODN and IR control groups).

In order to elucidate the role of death cascades, the expression of caspase-3 protein was measured. Caspase-3 interacts with caspase-8 and caspase-9, playing a central role in the execution-phase of cell apoptosis. Mice were exposed to 8 Gy of IR. Results showed that IR also induced caspase-3 expression in a position-dependent fashion (fig. 5A). The distribution of caspase-3 positive cell showed that for the irradiated control group, caspase-3 positive cell index was >5% at positions 3∼7, the index reached a maximum of 16.0% at position 5 and fall off rapidly to below 1%. For exposed mice treated with CpG-ODN, caspase-3 positive cell index was >5% only at position 5. The index reached a maximum of 5.5% at position 5 and fall off gradually to below 1% (fig. 5B). CpG-ODN reduced the number of caspase-3 positive cell at position 4 from 9.0% to 3.0% (fig. 5C). The results showed a 2.8-fold decrease in the number of caspase-3 positive cell for the irradiated group with CpG-ODN treatment compared with irradiated control group (fig. 5D). These results suggest that CpG-ODN down-regulated caspase-3 expression at position-dependent fashion after IR injury.

## Discussion

The replacement process of proliferating cells in the crypt maintains normal homeostasis of intestine, these proliferating cells replicate and differentiate in order to continuously and rapidly replace the terminally differentiated epithelial cell. IR-induced intestine injury from direct cytocidal effects on these proliferating cell and subsequent loss of the mucosal barrier, results in RIGS [3].

Under the present protocol, the protective effect of CpG-ODN on IR-induced intestine injury is manifested indirectly by significantly reduction of body weight loss (fig. 1A), improving mice survival (fig. 1B). The direct evidence showed the increase in the number of surviving crypt for the irradiated group with CpG-ODN treatment was about 32% compared to irradiated control group on day 7 after IR exposure (fig. 2B). After radiotherapy at the abdominal or pelvic region, a reduction of the intestinal crypt number is an important factor associated with the side effects that limit the dose delivered to patients. The RIGS syndrome is caused by the loss of a large amount of crypts. The 32% increase of survival crypts is expected to reduce the side effects observed after radiotherapy. It is generally known that the fate of the crypt is determined by the replacement of proliferating cells located within the crypt, including intestinal stem cells and proliferative crypt cells. If all cells die, the crypt is ‘sterilized’ and disappears. However, if one or more proliferating cells survive, they rapidly proliferate and regenerate the crypt with subsequent reconstitutions of the epithelium. After high dose of IR, most crypt cells undergo apoptosis, or stop proliferating temporarily or permanently, but a few proliferating cells still survive, playing a central role in the regenerating of crypts after IR injury. In particular, there are some stem cells which increase production of proliferative crypt cell, undergo rapid clonal expansion, follow by differential into the mature cells and eventually repopulate the mucosa [3], [22]. TUNEL results show that IR induce many crypt cell apoptosis, and the apoptotic response is highest (index >50%) for proliferating cells located at positions 3∼14, including stem cell located at cell position 4. However, treatment of CpG-ODN result in a 2.2-fold decrease in the number of apoptotic cell and the apoptotic response is highest for proliferation cells located at positions 5∼6, exclusive of stem cell (fig. 3B and fig.3 C). BrdU results show that a 1.9-fold increase in the number of proliferating cell for the irradiated group with CpG-ODN treatment compared with the irradiated control group (fig. 3F). From these results we conclude that CpG-OND maintains proliferating cell, especially stem cell, located within the intestinal crypt population and the regeneration after IR exposure.

Although the effect of CpG-ODN is not fully understood, one possible mechanism is inhibition of apoptosis. Apoptosis has been known as the main mode of crypt cell death after IR injury, which is modulated through the activation of a particular set of molecular proteins. Bcl-2 family molecules are major molecules of the caspase cascade on cell apoptosis after IR injury [18]. Bax is a pro-apoptotic cytoplasmic protein that translocate to the mitochondria in response to apoptotic stimuli. Bcl-2 is present in the outer the mitochondrial membrane and is thought to act as an anti-apoptotic protein at least partly by inhibiting the translocation of Bax as well as the release of cytochrome *c* from the mitochondria. [23], [24]. The study results show that there were less apoptosis for the irradiated group with CpG-ODN treatment than for the irradiated control group. Positional analysis indicated that the difference was largely confined to proliferating cell in intestinal crypt at positions 3∼14, approximately, where Bax expression was down-regulated and Bcl-2 expression was up-regulated. These conclusions suggest that CpG-ODN inhibiting IR-induced proliferating cells apoptosis is associated with the expression of Bax and Bcl-2. In an attempt to better characterize regulation effect of CpG-ODN on apoptotic activity in proliferating cell, the expression of caspase-3 was assessed. Caspase-3 is now considered a key biochemical hallmark of apoptosis. Its regulatory pathways converge on a common cell apoptosis effector called the caspase family and the activation of caspase [24]. The distribution and the number of caspase-3 positive cell were generally consistent with that of Bax positive cell. This result demonstrate that CpG-ODN inhibiting proliferating cell apoptosis may be involved to the regulation of cytochrome *c* from mitochondria, which interacts with Apaf-1 and subsequently activates caspase-3 via proteolytic processing. Although some key step in the cascade is not determined in the present study [25].

Overall, this study found that CpG-ODN treatment protects against IR-induced intestine injury. The treatment effect is mediated through enhancing intestinal crypt survival and maintaining proliferating cell located within the intestinal crypt population and regeneration. The mechanism might be that CpG-ODN inhibited proliferating cell apoptosis through regulating the expression of apoptosis-related protein, such as Bax, Bcl-2 and caspase-3. The signaling pathway may be involved to the regulation of cytochrome *c* from mitochondria, which subsequently activates caspase-3. A better understanding of the mechanisms related to the treatment effect of CpG-ODN on IR injury will require further studies.
